# Enhancing surveillance for dengue fever in Oyo State, Nigeria – a one health approach

**DOI:** 10.1186/s42522-024-00121-9

**Published:** 2025-01-29

**Authors:** Olawale Sunday Animasaun, Joseph Ojonugwa Shaibu, Busayo Kayode Akomolafe, Olamide Priscilla Animasaun, Piring’ar Mercy Niyang, Olukemi Titilope Olugbade, Akinfemi Oyewumi Akinyode, Ibukun Akinsola Omisakin, Adedapo Olufemi Adeogun, Rosemary Ajuma Audu

**Affiliations:** 1 Medical Virology Unit, Faculty of Basic Medical and Applied Sciences, Lead City University and Primary Health Care Board, Ibadan, Oyo State Nigeria; 2 Nigeria Field Epidemiology and Laboratory Training Programme, Abuja, Nigeria; 3 Georgetown Global Health Nigeria, Abuja, Nigeria; 4 Centre for Human Virology and Genomics, Nigerian Institute of Medical Research, Yaba, Lagos State Nigeria; 5 Department of Biochemistry, Federal University of Agriculture Abeokuta, Abeokuta, Nigeria; 6 Department of Public Health, Oyo State Ministry of Health, Ibadan, Nigeria; 7 Department of Medical Services, Ministry of Defense, 2 Division Nigeria Army, Ibadan, Oyo State Nigeria; 8 Department of Community Health, Obafemi Awolowo University Teaching Hospitals Complex, Ile-Ife, Nigeria; 9 Department of Medical Laboratory Science, McPherson University, Ogun State, Nigeria; 10 Department of Public Health and Epidemiology, Nigerian Institute of Medical Research, Yaba, Lagos State, Nigeria

**Keywords:** Dengue Fever, *Aedes* mosquito, Meteorological parameters, Surveillance, Diagnosis

## Abstract

**Background:**

Dengue fever (DF) poses a growing global threat, necessitating a comprehensive one-health approach to address its complex interplay between human, animal, and environmental factors. In Oyo State, Nigeria, the true burden of DF remains unknown due to underdiagnosis and misdiagnosis as malaria, exacerbated by poor health-seeking behavior, weak surveillance systems, and inadequate health infrastructure. Adopting a one-health approach is crucial to understanding the dynamics of DF transmission.

**Methods:**

A cross-sectional study was conducted from January 2022 to April 2023 in 10 high-risk LGAs of Oyo State. It involved screening DENV in 289 febrile human blood samples and 1,015 *Aedes species* mosquitoes. Viral RNA from human and mosquito specimens was extracted and analyzed using RT-qPCR. A one-step lateral flow immunoassay cassette test kit detected DENV-specific IgM and IgG in humans. DENV IgM-positive participants were screened for Lassa Virus (LASV) to rule out coinfection due to an outbreak of Lassa fever. Mosquitoes recovered were morphologically identified and classified using appropriate taxonomical keys. Meteorological data was obtained from the Nigeria Meteorological Agency. Data was abridged as proportions and correlation analysis was performed.

**Result:**

The overall seroprevalence of DENV was 128/289 (44.2%) with 19/289 (6.6%) and 109/289 (37.7%) being IgM and IgG positive respectively. DENV was detected all year round with more cases in the rainy season. LASV and DENV coinfection were detected in a participant. DENV RT-qPCR analysis in febrile patients and mosquitoes was negative. There was a high abundance of *Aedes aegypti (79.5%)* in all the locations surveyed with *Aedes albopictus (12.3%)* detected in Ido LGA and Ibadan South-East LGA and *Aedes simpsoni (9.1%)* in Iwajowa LGA. DF shows moderate to strong positive correlations with Aedes mosquito population, humidity, and rainfall (r = 0.419–0.61, *p* < 0.05), and a negative correlation with temperature (r =—0.465, *p* < 0.05).

**Conclusion:**

The study reveals a significant burden of DENV in Oyo State. The presence of both IgM and IgG antibodies suggests past exposure and possible recent circulation of the virus. The co-detection of LASV and DENV in one participant highlights the likely potential for co-infection. Although DENV was not detected in febrile patients and mosquitoes through RT-qPCR, the high abundance of Aedes species underscores the risk of transmission. These findings emphasize the need for enhanced surveillance systems, strengthened laboratory services, targeted vector control, and increased awareness.

## Introduction

Dengue fever (DF) is an arboviral infection caused by dengue virus (DENV) of the *Flaviviridae* family. It is transmitted to humans through bites of infected mosquitoes of *Aedes species*. Four related but antigenically discrete serotypes (serotypes 1–4) with 62—67% sequence homology of the DENV have been identified [1]. The classification of the four serotypes was based on the immunological response of patients to primary DENV infection by one of the serotypes. Primary infection of DENV protects against a secondary infection by a homologous DENV serotype but confers partial and transient protection against a heterologous DENV serotype [2]. Secondary exposure to a heterologous serotype can enhance the risk of DHF/DSS due to the phenomenon of antibody-dependent enhancement (ADE). ADE occurs when pre-existing antibodies from a previous infection with one serotype bind to a different serotype, enhancing viral entry into host cells and leading to increased viral replication. This can result in a more severe immune response, increasing the risk of dengue haemorrhagic fever (DHF) and dengue shock syndrome (DSS) which are severe and life-threatening forms of the disease. Furthermore, secondary infections with heterologous serotypes can also lead to a broader activation of immune cells, including T cells and macrophages, which can contribute to the development of DHF/DSS [3].

The incidence and magnitude of DF outbreaks have increased significantly as the virus itself and mosquito vectors have expanded geographically in tropical and subtropical regions. Consequently, 129 countries are at risk, with over 3.9 billion people and an estimated 96 million symptomatic cases with a projected 40,000 deaths each year [3]. Furthermore, in Africa and Asia, there is a high impending possibility for the re-emergence of sylvatic dengue in the human transmission cycle due to deforestation, population growth and encroachment to forests, climate change, and vector geographical expansion [4]. Dengue is a high-burden disease that disproportionately affects countries in the tropics and subtropics, many of which have limited healthcare resources [5].

In Nigeria, DF is endemic in almost all the states nationwide and could be the leading cause of unclassified febrile illnesses [5]. Dengue fever has a mixed distribution among urban, and rural areas and was previously predominantly reported in urban areas than in rural areas [5]. In Oyo State and Nigeria, DENV, YFV, and other arboviruses have a high potential to cause serious public health impact, due to the high density of the competent disease vectors, the existence of pockets of unvaccinated and susceptible populations due to low Yellow Fever (YF) vaccination coverage and non-availability of vaccines for other DF and other arboviral infections. Other limitations include epidemiological gaps slowing the detection of cases, non-availability of routine testing for DF as well as inadequate engagement of local healthcare providers in disease surveillance and management [6].

Central to the epidemiology of DENV and other arboviral infections, is the requirement for mosquito-borne transmission to primate hosts, these viruses are transmitted to man (and other vertebrates) by the bite of an arthropod vector, primarily mosquitoes. Despite the presence of the virus in the blood and bodily secretions during acute infection, DENV infection is not contagious. Consequently, available reservoirs of infectious viruses and high levels of vector populations are prerequisites for epidemic outbreaks. Having known this, Integrated vector management and control strategies, prompt case detection, and management are the main strategies for the prevention and control of dengue virus transmission.

Many arboviruses are circulating in Nigeria, YF for example, has caused large epidemics in the past. There is also serologic evidence of human infection with YF, DF, West Nile, and Zika virus. There are reports that many clinical arbovirus infections have been misdiagnosed as malaria and typhoid fever in the country [6]. DF endemicity in Oyo state and Nigeria in general is an ongoing phenomenon with its attendant burden on the health, economic, and socio-political spheres of the country. The magnitude of these is yet to be evaluated.

A seroprevalence study carried out in Osun state, southwest Nigeria, indicates that the IgG prevalence of DF is 77%. This indeed is very high, showing that this disease affects many people and is most likely responsible for a great proportion of febrile illnesses often misdiagnosed with malaria due to lack of routine diagnosis [4, 6].

The existence of several DENV serotypes in Nigeria indicates that large and repeated epidemics of DHF/DSS may happen in the future. DF surveillance is therefore crucial for the monitoring of the disease frequency or trend, and the discovery of outbreaks or new mutations, to trigger timely interventions. Outbreak alerts are essential to mobilize vector control and to prepare healthcare delivery services in readiness for a surge in frequency. This should be viewed in the context of the challenges encountered in the process of discovering and producing efficacious anti-DENV drugs and vaccines that are yet to be available.

Entomological investigations have confirmed the presence of the *Aedes aegypti* and *Aedes africanus* in several states across Nigeria [7]. These reflect the activities of several competent mosquito vectors, capable of transmitting arboviral infections from reservoirs to other hosts in the country [8]. Some of these mosquito vectors have been implicated following past arbovirus epidemics. Knowledge of the species composition and fluctuations that take place in mosquito vector populations in response to abiotic environmental conditions, principally weather, is necessary to identify periods when these vectors thrive the most and when arboviruses are at their peak of transmission. A multidisciplinary and multisectoral collaboration, through a One Health approach which this study was designed to achieve, is therefore required to effectively prepare, detect, assess, and respond to emerging and endemic arboviral diseases. In this study, we investigated the public health burden, meteorological influence, high-risk groups, abundance of *Aedes* mosquitoes, and DENV carriage rate of *Aedes* mosquitoes in Oyo State.

## Materials and methods

### Study area

Oyo State is in the South-West geopolitical zone of Nigeria with Ibadan city as the capital. This State has 33 Local Governments Areas (LGAs) and 29 Local Council Development Areas (LCDAs) and a projected population of 9,233,010 with an annual growth rate of 3.4 according to the National Population Commission 2019 report. The dry season lasts from November to March characterized by low relative humidity, and high environmental temperatures with low or no rainfall while the wet season starts from April and ends in October with high relative humidity, lower environmental temperatures, abundant rainfall, and often flooding [1]. Average daily temperature ranges between 25 °C (77.0 °F) and 35 °C (95.0 °F), almost throughout the year. The vegetation pattern of Oyo State is that of rainforest in the south and guinea savannah in the north. Thick forest in the south gives way to grassland interspersed with trees in the north [9].

### Study location

**Fig. 1:**
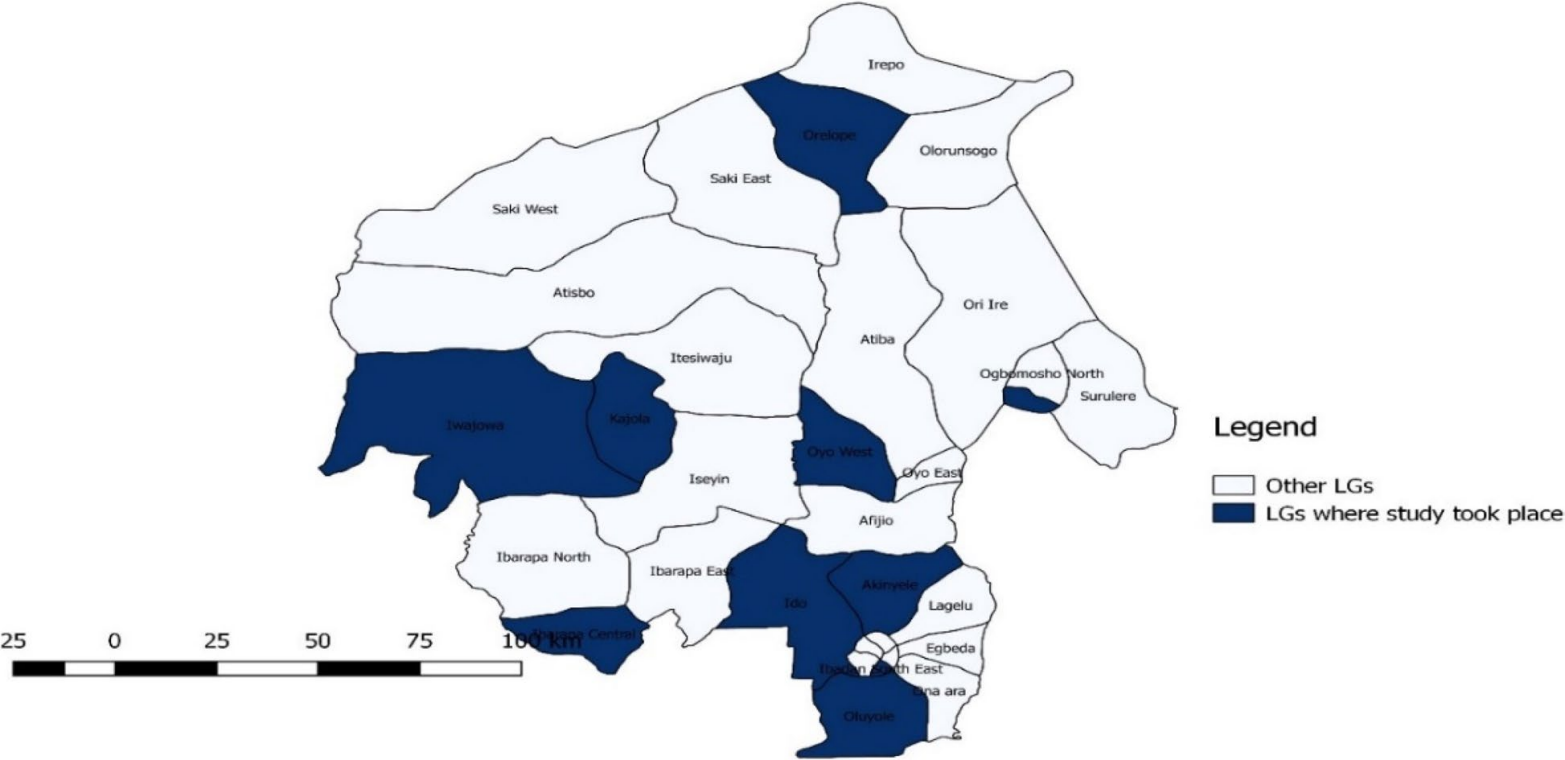
Map of Oyo State, Nigeria showing study locations

Human and entomological surveillance took place in 10 high-risk LGAs in Oyo State where confirmed cases of an arboviral or viral haemorhagic infection(s) had been reported by researchers, the State Ministry of Health and the Nigerian Centre for Disease Control (NCDC). The LGAs were as follows: Ido, Oyo East, Orelope, Ona-Ara, Akinyele, Oluyole, Iwajowa, Ogbomosho South, Ibarapa Central, and Ibadan Southeast (Fig. 1).

### Study design

This was a cross-sectional study that involved human and entomological surveillance for DENV. The duration of the study for human sampling was January 2022 to April 2023 while the entomological surveillance took place from May 2022 to April 2023.

### Study population

This is a mixed population study as participants were drawn from febrile in and out-patients attending selected healthcare facilities, some were recruited through active case search in communities involving house-to-house case finding, and some enrolled through reporting to the State emergency operation center via the integrated disease surveillance system strategy in the selected LGAs.

### Case definitions

#### Suspected DF case

Is “anybody with acute febrile illness of 2–7 days duration with at least one or more of the following symptoms: headache, myalgia, arthralgia, rash, vomiting, anorexia, haemorhhagic manifestations and, leucopenia.

#### Confirmed DF case

A suspected case with laboratory-confirmed (positive IgM antibody, rise in IgG antibody titre, positive PCR or viral isolation).

Febrile patients with confirmed diagnoses of other infections, those outside study areas, and non-consenting patients were excluded from the study.

### Sampling techniques

A purposive sampling technique was used in the enrollment of study participants for this study based on meeting the case definitions.

### Data collection techniques

A quantitative approach using structured tools was adopted for data collection. Semi-structured interviewer-administered questionnaires were used to obtain information from selected consenting participants.

### Sample collection, transportation, and storage

Five (5) mL of whole blood was aseptically collected via venipuncture into an ethylene diamine tetraacetic acid anticoagulant bottle and transported from the field to the laboratory using a triple packaging system in a geostyle at + 2[0]C to 4[0]C. The samples were centrifuged for 5 min at 3000 g to obtain the plasma which was aliquoted into two parts for serology and molecular testing and then stored frozen at -20[0]C.

### Laboratory analysis

All laboratory processes were performed at the Center for Human Virology and Genomics, Nigerian Institute of Medical Research (NIMR), Yaba, Lagos, Nigeria.

#### RNA extraction process

Following the manufacturer’s instructions, RNA was extracted from human plasma and mosquitoes (10 per poll were mechanically crushed) using the Jena Bioscience Viral DNA + RNA purification Kit (Jena, Germany). Extracted RNAs were Stored at -20 °C for further processing.

#### Real-time PCR analysis

One-step reverse transcriptase (RT) real-time (qPCR) was carried out to detect the pathogen using a Quant Studio5 machine. The amplification steps encompass denaturation, primer annealing, and elongation. Optimization of the PCR conditions was done for optimum amplification of the genes. Positive and negative controls were set up and run along with the test. ATCC panels (DENV types 1–4) and a total RNA extract from a previous study were used as positive controls [10]^.^

#### Cycling conditions

Initial denaturation at 94˚C for 5 min, then by 36 cycles of denaturation at 94˚C for 30 s, annealing at 55˚C for 30 secs, and elongation at 72˚C for 45 s. Followed by a final elongation step at 72˚C for 7 min and hold temperature at 10 ˚C.

#### PCR mix components

The PCR mix was made up of 25 µl containing 10 µl enzyme mix, 0.8 µl of forward and reverse primers; 0.4 µl for probes, 6 µl of nuclease-free water, and 5 µl of RNA template was set up. A Ct value of < 38 was adjudged to be positive for viral pathogens.

### Primer and probe used for the detection of DENV

Forward5’ AAACCGCGTGTCGACTGTGC 3’.

Reverse3’TAGGAAACGAAGGAATGCCACC5’.

ProbeFAM-5’CACTTGGAATGCTGCAGGGACGAGGACC3′ [10].

### Serological analysis

A one-step lateral flow immunoassay cassette test kit from Zijian Biotechnology, Shenzhen, China was used following the manufacturer's instruction for the detection of Dengue Virus-specific IgG and IgM. Dengue IgM/IgG Rapid Test Kit (ZJ-001): Sensitivity: 92.5% (IgM), 95.5% (IgG) and Specificity: 96.5% (IgM), 97.5% (IgG) while ReLASV® Pan-Lassa NP IgG ELISA Kit (Human anti-LASV NP Antibody) was used for detection of IgM antibodies against LASV for samples found positive to DENV Ig M. The specificity and sensitivity of the ELISA Kit are 92.6% (95% CI:87.5–96.5%), and 96.4% (96% CI:93.50–98.5%) respectively.

## Entomological surveillance

### Vector trapping, identification, transportation and processing

**Fig. 2:**
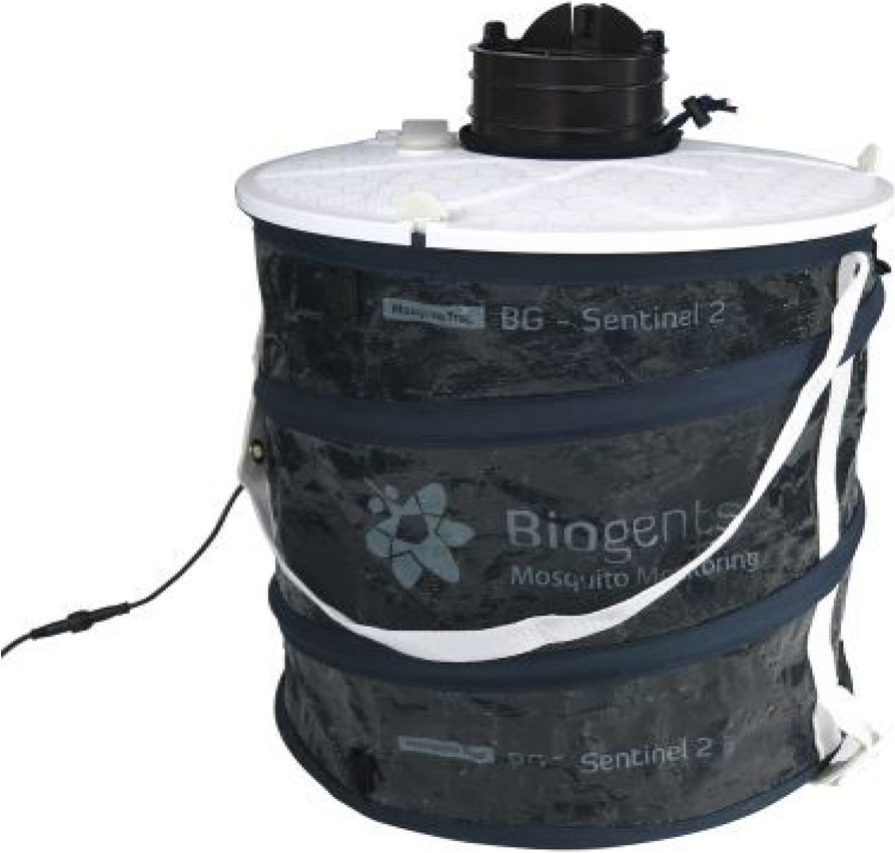
Biogent mosquito trapping device

Two entomological approaches were employed in the survey: the Larval/Pupae survey and the use of Biogents Sentinel Trap (BG Trap) for adult mosquitoes. The Mosquitoes were sampled in all months- rainy and dry seasons using traps at different sampling plots per site. Each trap was deployed at randomly selected locations with high anthropogenic activities during a sampling event. The same efforts were replicated in all our sampling plots during the study periods. The sampling of mosquitoes broadly targets *Aedes* species as studies have implicated its role in the transmission of a variety of viruses [11] (Fig. 2).

The BG trap mimics convection currents created by the human body, it employs attractive visual cues and releases artificial emanations through a large surface area. It has a BG lure, a dispenser that releases a combination of non-toxic substances that are also found on human skin (ammonia, lactic acid, and caproic acid). The BG-Sentinel mosquito trap is essentially a collapsible, fabric container with a white lid with holes covering its opening. The diameter is 36 cm (14 inches), the height is 40 cm (1.3 feet). In the middle of the gauze cover, the air is sucked into the trap through a black catch pipe by an electrical fan, drawing approaching mosquitoes into a catch bag.

The air then exits the trap through the white gauze, generating ascending currents (red arrows). These are like convection currents produced by a human host, both in its direction, its geometrical structure, and, due to the addition of the BG-Lure, also in its chemical composition. Mosquitoes were caught using prokopack aspirators and test tubes.

Larva sampling was also conducted targeting the immature stages of domestic and peri-domestic breeders among the *Aedes* species. Natural habitats such as leaf axils, which might harbour immature stages (larvae and pupae) of *Aedes* species were inspected to determine their presence or absence. Likewise, artificial containers in and around various houses were inspected for larvae and pupae of the vectors. Where possible, the entire water content of a container was emptied into a bowl and the immature stages were picked using pipettes. They were then introduced into well-labeled containers and taken to the entomological laboratory of University of Ibadan, where they were breaded and allowed to develop to adults for proper identification.

### Mosquito identification and preservation

Adult mosquitoes collected in the field and those reared to adults from immature collections were euthanized by freezing at -20º C for 20 min. The specimens were morphologically identified to species level using appropriate taxonomic keys, they were sorted out and identified by morphological characteristics with the key aids of Leopoldo M. Rueda [11]. They were later counted, recorded and introduced into well-labeled Eppendorf tubes containing RNA later(shield) and then stored frozen at -20[0]C.

#### Inclusion criteria

To improve the chances of successful viral yield from the mosquitoes, live mosquitoes were trapped in high anthropogenic locations.

#### Exclusion criteria

Dead mosquitoes were not used. Also, those not in designated sample locations.

### Determination of mosquito larval indices

The following larval indices were used to determine the risk of transmission:

**House Index** (HI; percentage of houses with at least one positive container) i.e.

$$\frac{Number\;of\;houses\;infested\;with\;Aedes\;larvae\;and/or\;pupae}{Number\;of\;houses\;sampled}\times100$$

**Container Index** (CI; percentage of all containers with water that are larva and/or pupa positive) i.e.

$$\frac{Number\;of\;containers\;positive\;for\;Aedes\;larvae\;and/or\;pupae}{Number\;of\;containers\;sampled}\times100$$

**Breteau Index** (BI; the number of positive containers per 100 houses) i.e.

$$\frac{Number\;of\;containers\;positive\;for\;Aedes\;larvae\;and/or\;pupae}{Total\;number\;of\;houses\;sampled}\times100$$

### Meteorological parameters

The data on minimum and maximum temperature, rainfall, and relative humidity between May 2022 and April 2023 were collected every month from the Nigeria Meteorological Agency (NIMET). Temperature is defined as the average daily temperature (°C) measured using temperature loggers, rainfall is defined as the total monthly rainfall (mm) measured using rainfall gauges, and relative humidity is defined as the average daily relative humidity (%), measured using hygrometers.

### Data management and analysis

Data was analyzed using Epi info Statistical package Version 7.0 and Microsoft Excel. Epi Info was used for data entry, cleaning, and basic analysis, while correlation analysis was performed using Microsoft Excel. Quantitative data was presented using tables and charts. Correlation analysis was used to test the association between Dengue Fever, *Aedes* mosquito vector abundance, and environmental factors (temperature, rainfall, and relative humidity). We used r > 0.3 to show a moderate association and r > 0.5 as a strong association.

## Results

### Sociodemographic of study participants

A total of 289 febrile patients were enrolled in this study, there were more female respondents 164/289 (56.7%) and the age group 30–44 years constituted the majority 106(36.7%) (Table 1). Clinical symptoms among the DENV-positive cases were high-grade fever > 38 °C 19/19 (100%), fatigue 12/19 (63.2%), and jaundice 2/19 (10.5%).

**Table 1:** Socio-demographic characteristics of participants in Oyo State January 2022-April 2023

Variables	Frequency(*N* = 289)	Percentage (%)
**Sex**		
Male	125	43.3
Female	164	56.7
**Age group in years**		
0–14	41	14.2
15–29	58	20.0
30–44	106	36.7
45–59	54	18.7
Above 60	30	10.4
**Educational level**		
No formal education	96	33.2
Primary	105	36.3
Secondary	58	20.0
Tertiary	30	10.4
**Profession/Employment status**		
Civil servant	45	17.0
Farming	59	20.4
Cattle Breeders	80	27.7
Self-employed	47	16.3
Trading	28	9.7
Unemployed	10	3.5
Students	22	7.6

### Molecular results of human and mosquitoes samples

RT-qPCR analysis of the RNA extracted from all human blood samples collected from our study participants and mosquitoes’ samples were negative for DENV RNA.

### Serological results

**Fig. 3:**
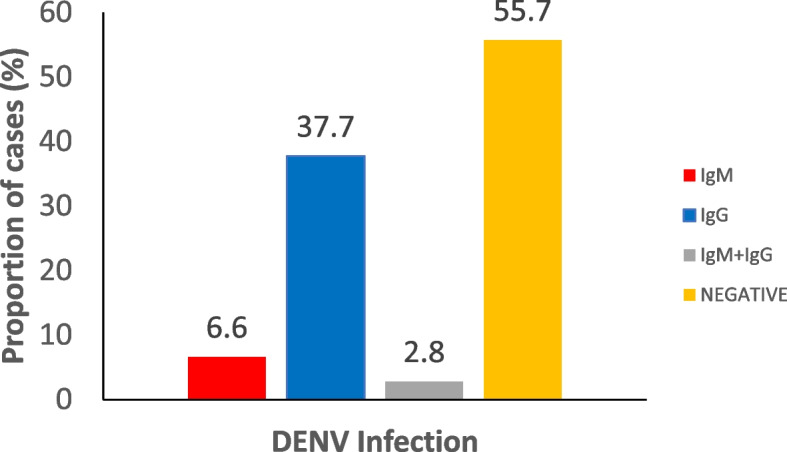
Seroprevalence of DENV in Oyo State, Nigeria

The overall seroprevalence of DENV in Oyo state was 128/289 (44.3%) consisting of 19/289 (6.6%) and 109/289 (37.7%) IgM and IgG positive, 8/289 (2.8%) of the participants were simultaneously positive for IgM and IgG specific DENV (Fig. 3).

**Fig. 4:**
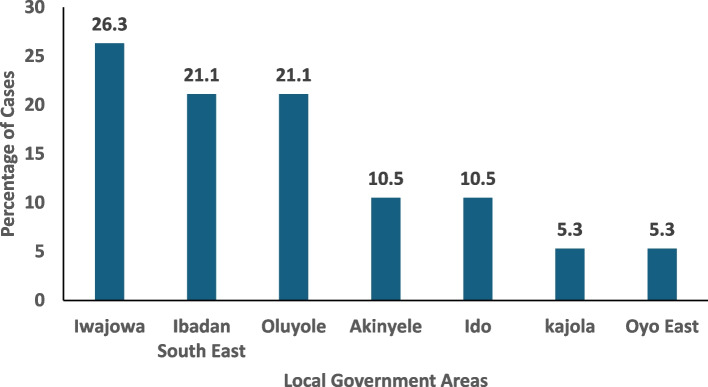
Distribution of DENV IgM-positive persons by Local Government Area in Oyo State, Nigeria

**Fig. 5:**
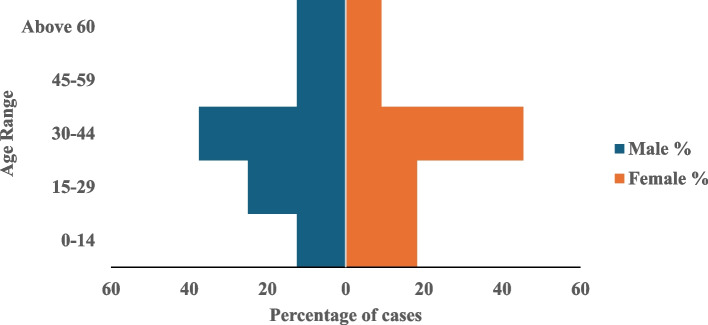
Sex and age distribution of DENV IgM confirmed cases in Oyo State from Jan 2022 to April 2023

**Fig. 6:**
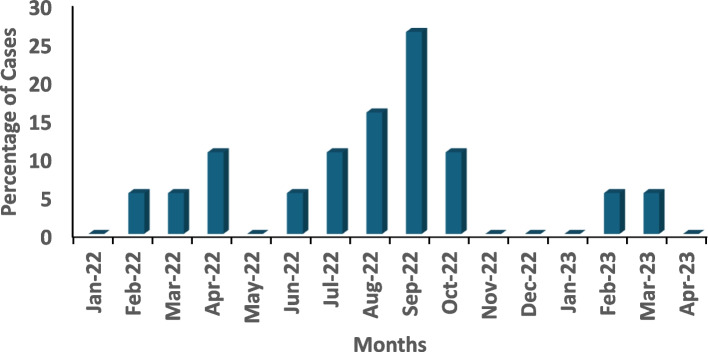
Monthly distribution of DENV IgM confirmed cases in Oyo State from January 2022 to April 2023

**Fig. 7:**
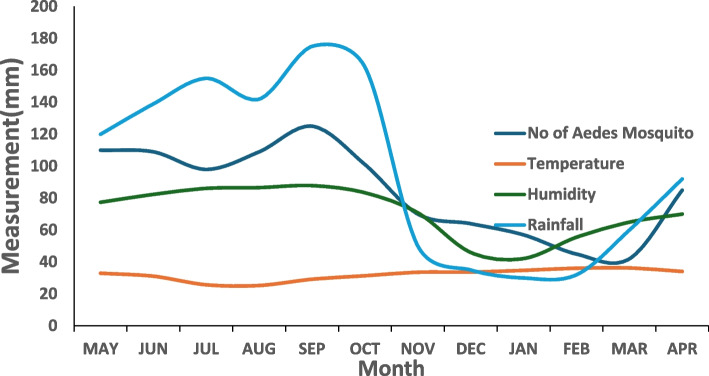
Monthly abundance of Aedes species with environmental factors (Temperature, Humidity, and Rainfall) in Oyo State Nigeria, May 2022 to April 2023

The distribution of DENV-positive IgM patients by LGAs (Fig. 4) showed five (26.3%) patients from Iwajowa LGA, four (21.1%) from Ibadan Southeast and Oluyole LGAs, two (10.5%) from Ido and Akinyele LGAs, with one person (5.3%) in both Kajola and Oyo East LGAs were identified Females 11/19(57.9%) and the age group 30–44 years were the most affected (Fig. 5). A Patient was positive for both LASV IgM and DENV IgM. In this study, DENV IgM positive cases were reported almost all year round with more cases reported in the months of the rainy season July to October (Fig. 6). Also, there exists a trend in the abundance of *Aedes* mosquitoes recovered with an increase in humidity and rainfall (Fig. 7).

### Larval survey and adult Aedes species composition

A total of 776 containers/tires were inspected from houses in 10 different communities in 10 LGAs of Oyo state, of these numbers, 254/500 (50.8%) of the houses had positive Aedes larvae/pupae and 361/776 (46.5%) of water retaining containers/tires had various positive immature stages of Aedes species (Table 2)*.* Adult Aedes mosquitoes were collected using BG trap, a total of 414 were recovered. The BG trap mimics convection currents created by a human body, it employs attractive visual cues and releases artificial emanations through a large surface area. It has a BG lure, a dispenser that releases a combination of non-toxic substances that are also found on human skin (ammonia, lactic acid and caproic acid)*.*

**Table 2:** Household and container productivity of Aedes species per community *in Oyo State from May 2022 to April 2023*

S/N	LGA	Location / Community	Number of Houses Surveyed	Number of Houses with Positive Aedes Pupae/Larvae	Number of Containers/Tires Surveyed	Number of Containers with Positive *Aedes* Larvae/Pupae
1	Ido	Leo	50	23	94	41
2	Oyo East	Araromi	50	34	75	32
3	Orelope	Igboho	50	15	50	14
4	Ona Ara	Kajola	50	16	92	18
5	Akinyele	Olorisaoko	50	32	76	48
6	Oluyole	Ire-Akari	50	28	71	45
7	Iwajowa	Agbaruru	50	35	80	40
8	Ogbomoso South	Arowomole	50	25	49	28
9	Ibarapa Central	Igboora	50	21	67	37
10	Ibadan South East	Oranyan	50	25	122	48
	**10 LGAs**	**Total**	**500**	**254 (50.8%)**	**776**	**361 (46.5%)**

**Fig. 8:**
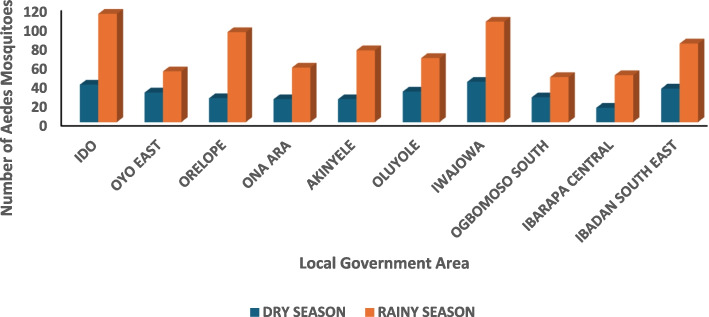
Seasonal abundance of all Aedes mosquitoes in selected LGAs in Oyo state from May 2022 to April 2023

There was a high abundance of *Aedes aegypti* in all the locations surveyed with fewer quantities of *Aedes albopictus* detected in Ido LGA and Ibadan South-east LGA while *Aedes simpsoni* was found at Iwajowa LGA (Table 3)*.* The total Aedes species collected during the study period was 1015, consisting of *Aedes aegypti* 798/1015(78.6%)*, Aedes albopictus* 95/1015(12.3), *and Aedes simpsoni* 62/1012(9.1%) (Table 4). Anopheles and other species also recovered from the 10 different communities in abundance. The number of mosquitoes recovered all over the study location during dry season was in the same range, there was however an appreciable increase all over the LGAs with Ido and Iwajowa having the highest Aedes mosquito abundance in the rainy season followed by Ibadan South-East as seen in Fig. 8.

**Table 3:** Aedes(larval) species composition *in Oyo State from May 2022 to April 2023*

S/N	LGA	Community	Aedes spp	Number	Total No
1	Ido	Leo	*Ae. aegypti* *Ae. albopictus*	89 33	122
2	Oyo East	Araromi	*Ae. aegypti*	54	54
3	Orelope	Igboho	*Ae. aegypti*	69	69
4	Ona Ara	Kajola	*Ae. aegypti*	40	40
5	Akinyele	Falade- Olorisaoko	*Ae. aegypti*	39	39
6	Oluyole	Ire-Akari	*Ae. aegypti*	47	47
7	Iwajowa	Agbaruru	*Ae. aegypti* *Ae. simpsoni*	82 25	107
8	Ogbomoso South	Arowomole	*Ae. aegypti*	21	21
9	Ibarapa Central	Igboora	*Ae. aegypti*	19	19
10	Ibadan Southeast	Oranyan	*Ae. aegypti* *Ae. albopictus*	71 12	83
			**Total**	601

**Table 4:** Relative abundance of all Aedes (Adult and Larval) collected in Oyo State, Nigeria from May 2022 to April 2023

S/N	Aedes Species	Number	Proportion
**1**	*Aedes aegypti*	798	78.6
**2**	*Aedes albopictus*	125	12.3
**3**	*Aedes simpsoni*	92	9.1
	Total	**1015**	**100**

The correlation coefficients r = 0.419166*,* r = 0.568597, and r = 0.601639 as seen in Table 5 all indicate a positive association between Dengue Fever vs No of Aedes Mosquito, Humidity, and Rainfall respectively while r = -0.46593 shows a negative association between Dengue Fever vs temperature. The larval indices were high in all the communities sampled as seen in Table 6

**Table 5:** Correlation between DENV, Mosquitoes, and environmental factors (Rainfall, Humidity, and Temperature) Oyo State, Nigeria from May 2022 to April 2023

	Dengue	No of Aedes Mosquito	Temperature	Humidity	Rainfall
Dengue	1				
No of Aedes Mosquito	0.419166*	1			
Temperature	-0.46593	-0.74949	1		
Humidity	0.568597*	0.827154	-0.73563	1	
Rainfall	0.601639^*^	0.90958	-0.77836	0.923878	1

Interpretation: correlation coefficient r = 0.419166 *p*-value < 0.05, r = 0.568597 *p*-value < 0.05, r = 0.601639 *p*-value < 0.05. r> 0.3 shows a moderate association and r> 0.5 shows a strong association between Dengue Fever vs No of Aedes Mosquito, Humidity, and Rainfall respectively while r= -0.46593 *p*-value < 0.05 shows a negative association between Dengue Fever vs temperature

These results suggest that as the number of Aedes Mosquito, humidity, and rainfall increase, the incidence of Dengue Fever also tends to increase. In contrast, as temperature increases, the incidence of Dengue Fever tends to decrease

**Table 6:** Larval indices Aedes collected in Oyo State from May 2022 to April 2023

S/N	Local Government	House index (%)	Container index (%)	Breteau Index (%)
1	Ido	46	43.6	82
2	Oyo East	68	42.7	64
3	Orelope	30	28	28
4	Ona Ara	32	19.6	36
5	Akinyele	64	63.2	96
6	Oluyole	56	63.4	90
7	Iwajowa	70	50	80
8	Ogbomoso South	50	57.1	56
9	Ibarapa Central	42	55.2	74
10	Ibadan Southeast	50	39.3	96

Generally, according to WHO guidelines, the larval index is considered low when the House Index ˂ 5% and the Breteau Index ˂ 4. It is high when the House Index ≥5% and/or the Breteau Index ≥4 [3]. All locations surveyed fall into WHO Priority 2 (i.e. localities with high larval indices (House Index ≥5% and/or Breteau Index ≥4)

## Discussion

The concept of One Health has evolved significantly since its inception. A more comprehensive definition, supported by the Quadripartite organizations the Food and Agriculture Organization of the United Nations (FAO), the United Nations Environment Programme (UNEP), the World Health Organization (WHO), and the World Organization for Animal Health (WOAH), is essential to reflect the current understanding. One Health is defined as the collaborative effort of multiple disciplines working locally, nationally, and globally to attain optimal health for people, animals, and the environment. This paradigm recognizes the interrelatedness and interdependence of human, animal, and ecosystem wellbeing, emphasizing the need for systematic and cross-sectoral approaches to address global public health emergencies and health threats at the human-animal ecosystem interface. This study adopted the One Health approach to achieve its objectives, which included determining the sero-molecular prevalence of DENV, assessing Aedes mosquito abundance and its arboviral carriage rate, and examining the influence of climatic and meteorological parameters on Aedes mosquito vector abundance in Oyo State, Nigeria. By embracing the One Health concept, this research demonstrates the value of interdisciplinary collaborations and communications in understanding the complex relationships between human, animal, and environmental health.

In Nigeria, there is growing evidence consequent upon lessons learned from the response to previous outbreaks that no single sector/agency can sufficiently manage the challenges of myriads of public health threats battling the nation [12]. Experiences from the Lassa fever, Cholera, COVID-19 outbreaks” responses, and ongoing activities in combatting antimicrobial resistance have demonstrated the effectiveness of multi-sectoral, multi-agency approaches in dealing with public health issues.

Consequently, the One Health approach has been advocated as the global framework for strengthening collaboration and capacities of the sectors and actors involved in health service delivery [12].

### Dengue fever sero-molecular detection

Before this study, the magnitude of DF in Oyo state and indeed in Nigeria remained unclear, as cases were underdiagnosed or misdiagnosed as malaria or referred to as pyrexia of unknown origin (PUO) if they failed to respond to antimalarial drugs [6, 13]. The high detection rate (44.3%) of DENV among participants in this study may arguably make DF responsible for some unclassified febrile illnesses; misdiagnosed as Malaria or as Pyrexia of Unknown Origin. This is particularly so because DF is usually not a differential diagnosis of acute febrile illness hence, testing is not usually requested [14]. There is currently no ongoing surveillance activity dedicated to DF in Oyo state and the surveillance activity for DF in the NCDC needs to be strengthened. What exists are seroprevalence and molecular studies carried out by individual researchers in various parts of the country which have confirmed high prevalence and transmission of DF in Nigeria [5, 6, 13]. Though DF has been declared a priority epidemic-prone disease, the reciprocal surveillance activity including testing of suspected cases or inclusion as differential testing for other VHF is missing.

There are no sufficient morbidity and mortality data available on DF now in Oyo state and Nigeria at large. In this study, DENV IgG was used to assess past exposure to infection, while DENV IgM to assess active or acute infection. The seroprevalence of 37.7% for DENV IgG was reported in this study which is in close range with the overall pooled DENV IgG prevalence of 32.8% reported by a systematic review and lower than an IgG seroprevalence of 77.0% previously reported by a study conducted in Osun State [4, 13].

The DENV IgM seroprevalence of 6.6% reported in this study is lower than the IgM pooled seroprevalence of 16.8% reported by the same systematic review study and another study conducted previously conducted in Oyo State which reports IgM seroprevalence of 17.2% [9, 13].

The DENV-positive IgM patients were seen mostly from Iwajowa LGA followed by Ibadan Southeast, Oluyole, Ido, Akinyele, and Kajola LGAs, of the state. The relatively high prevalence of DENV in some of these LGAs could be due to the proximity of the localities to the dense rainforest which provides enabling breed sites for DENV vectors. This study also confirmed a high abundance of Aedes mosquitoes in the localities. More so, the primary host of the DENV, which is the non-human primates (NHP), is abundantly found in the forests [15]. This shows that this disease affects many people in Oyo state and is most likely responsible for a great proportion of febrile illnesses often misdiagnosed as malaria. This study shows that the state is a high burden state for DENV and there is an urgent need to include DENV for routine diagnosis [16].

The DENV RT-PCR for all samples tested were negative, this may be because DENV RNA is usually detectable in infected individuals a few days after infection till the sixth-day post-onset of clinical symptoms such as fever and headache [5, 17]. In Nigeria where health-seeking behavior is poor and individuals tend to consult traditional, patent medicine vendors ahead of presenting at health care facilities, and even when such patients come to the health facility, there is usually no routine laboratory testing capacity to screen for DENV, in such occasion DENV is usually missed out hence, it is possible that the samples were not obtained during the high infectious period [18–20].

DENV IgM is measurable from the third-day post-symptom onset till the third month, while IgG is detectable from day 10 post-onset of symptoms and can remain detectable for months and years [21]. Also, the exact duration of DENV RNA detectability varies depending on factors like viral load, individual immune response, and sampling methods. Hence, the serology test is suitable for diagnosis in this context, however, cross-reaction with other arbovirus and other limitations should be noted. In this study, some demographic and ecologic factors in the study location facilitated the spread of the DENV [22]. The increase in DENV antibodies with age from adulthood suggests that infection occurs from middle to older age having an age range 15–29 followed by 30–44 with the highest proportion of DENV. Although seropositivity was higher in female participants, these results were not significant, suggesting that both sexes are equally infected in this region. More so, a high level of IgG with a low level of IgM suggests infection in the past, thereby confirming the endemicity of DENV infection in the state.

IgG antibodies against DENV can persist for decades after a single infection, leading to a cumulative effect with age, multiple exposures to DENV can result in a boost in IgG levels, which can also contribute to the observed age-related increase. Studies have shown that IgG levels against DENV increase with age, even in the absence of recent exposure, due to the long-term persistence of antibodies [17, 20]. However, It is important to note that vaccination-induced immunity is a deliberate intervention aimed at inducing protective immunity, whereas natural exposure may not always result in protective immunity hence the observed IgG seroprevalence reported in this study raises our concern for past and recurrent exposure as no vaccination programme is in place in the nation targeting DF.

The high seroprevalence of DENV observed in this study may therefore indicate the poor level of public health interventions aimed at effectively controlling the vector transmission as well as interruption of the disease process. There is also the possibility of poor awareness of the presence of this disease in Oyo state and Nigeria as a whole, hence, the reason for the weak surveillance and routine testing. The high rate of urbanization in Oyo state equally explains the high prevalence of DENV, especially in urban LGAs within the Ibadan metropolis as the burden of DENV infection has been reported to be associated with the rising rate of urbanization, coupled with poor infrastructure in tropical and subtropical areas [12]. The mechanism behind the association between the high prevalence of DENV infection could be due to the high vector population and breeding sites because of urbanization and deforestation as seen in this study.

The high number of these breeding sites is favoured by climatic as well as environmental patterns of Nigeria that encourage the development of mosquitoes hence, favouring enhanced DENV transmission [11, 12]. Indeed, Aedes aegypti thrive better in warm and humid climatic conditions which typically exist in the study area [7]. This favours the survival of the mosquito eggs compared to the cold and temperate climatic regions which encourage hibernation while suppressing conservation and development [23, 24]. In this study, cases of DENV were reported almost all year round with more cases reported in the rainy season of July to October. There also exists a positive and strong correlation between DF, humidity, and rainfall, this further demonstrates the high prevalence of arboviral disease in the rainy season. It will be of interest to conduct a similar study in the northern part of the country with lower rainfall and humidity and higher temperatures.

### Entomological findings and detection of DENV in *Aedes* mosquito

Entomological findings from this study revealed a high abundance of *Aedes aegypti* in all the locations surveyed with few numbers of *Aedes albopictus* and *Aedes simpsoni* detected at Ido, Ibadan Southeast, and Iwajowa LGAs respectively. This was observed all year round with month-by-month variation in mosquito abundance, mainly caused by rainfall as established by this study. A similar study conducted in Ekiti reported high recovery of Aedes aegypti which was attributed to rainfall [7]. The all-year-round presence of Aedes species may be because their larvae can colonize and survive in almost all habitats such as barrels, drainages, tyres, pots, discarded plastics and bottles, and tanks from which they were trapped [7, 8, 11].

This study also revealed high larval indices as deduced from all the locations surveyed [3]. This is a surrogate marker and a clear indication that those communities are at high risk of massive DF outbreaks and other arboviral diseases, in the presence of a susceptible human population and competent disease vector [25]. To mitigate this, herd immunity to the prevalent arboviral disease through reactive mass vaccination for the vaccine-preventable arboviral disease is advocated as well as massive, targeted vector control. Given these, Oyo state is at risk of DF and other arboviral transmission, therefore multifaceted response actions are advocated. In essence, many Aedes mosquito species collected in this study are known to be important vectors of arboviruses of public health importance. Furthermore, in this study, high Aedes species abundance was found in months with high rainfall quantities with the peak in July and August while the lowest number was in February when there was little or no rain which agrees with a previous study done in Ibadan [21]. Mosquito populations varied during the study period in response to prevailing weather conditions. This research demonstrates that changes in rainfall and relative humidity due to imminent climate changes and seasonal pattern variations modify the behavior geographical distribution and abundance of Aedes mosquito vectors. Rainfall increases the opportunity for egg-laying by increasing the number of potential breeding sites for Aedes mosquitoes to lay eggs, which can reach adulthood within nine to twelve days, necessary for the mosquito life cycle. Rainfall is one of the climatic variables that aid in the multiplication of mosquito breeding places while humidity improves mosquito survival rates. The rainy season is a fertile period for breeding sites, which are numerous. Meteorological parameters are therefore good predictors of an abundance of Aedes mosquitoes and their associated disease risks [8, 13].

### Coinfection of LASV and DENV

So far, the co-infection of DENV and LASV has not been well documented in Nigeria. In this study, both LASV and DENV were simultaneously laboratory-confirmed in a 45-year-old male from Iwajowa LGA. The patient was positive for both LASV IgM and DENV IgM, this is like findings reported in Brazil which demonstrates co-infection of SARS-COV-2 and DENV [26]. This was the first case of human co-infection of DENV and LASV reported in Oyo State, Nigeria. This underscores the importance of an accurate and timely diagnosis for targeted and effective intervention.

### Contributions to knowledge

This study has provided useful information on the serostatus and ongoing transmission of DF in Oyo State and suggests the potential for a massive outbreak if the trends continue. Also, the Aedes species vector abundance in Oyo State has been established as well as the association between DF transmission, vector abundance, and climatic conditions.

### Study limitations

1. This study primarily focused on the human, vector, and environmental aspects. It did not incorporate the sylvatic circulation of dengue in forests, including other hosts beyond humans, to fully embody the comprehensive One Health approach advocated by the quadripartite (WHO-WOAH-FAO-UNEP), we at this moment recommend further study in this aspect with wildlife expert to provide a more complete understanding of the disease's ecology.
2. Cell culture of mosquito lysates to amplify dengue virus was not done in this study, this is because the mosquitoes were immediately preserved in an RNA shield (a reagent used to protect RNA integrity), which can potentially affect mosquito cell culture in several ways ultimately inhibiting cell growth, we recommend further study involving mosquito cell culture.

## Conclusion

In conclusion, this study underscores the critical importance of the One Health approach in addressing the multifaceted challenges posed by arboviral diseases such as DF in Oyo State and Nigeria in general. The findings reveal a high seroprevalence of DF in the region, indicating significant ongoing transmission and exposure. The serological data highlights the need for improved surveillance and diagnostic practices, as DF is frequently misdiagnosed due to its similarity with other febrile illnesses such as malaria. The high prevalence of Aedes mosquitoes, particularly Aedes aegypti, coupled with the observed correlations between mosquito abundance and climatic factors, reinforces the role of environmental and ecological factors in disease transmission. The study's detection of co-infection with Lassa fever virus (LASV) further emphasizes the complex interplay between different pathogens and the necessity for accurate and timely diagnoses to guide effective interventions. The study also identifies gaps in current public health strategies, including the absence of routine DF surveillance and the need for enhanced vector control measures. Given the high burden of DF and the presence of competent mosquito vectors, a multi-faceted approach involving improved surveillance, targeted vector control, and public awareness campaigns is crucial. Moreover, while this research provides valuable insights, it also highlights limitations, such as the lack of a comprehensive evaluation of sylvatic dengue circulation and the challenges associated with mosquito cell culture. Future studies should address these gaps to offer a more complete understanding of the disease ecology and improve control measures. Overall, the adoption of the One Health framework is vital for effectively managing and mitigating the risks of DF and other arboviral diseases. By fostering interdisciplinary collaboration and integrating environmental, animal, and human health perspectives, stakeholders can develop more robust strategies to combat the spread of infectious diseases and enhance overall public health outcomes.

## Data Availability

The datasets used and or analyzed during this current study are available from the corresponding author on reasonable request. Relevant data generated and analyzed during this study are included in the published article.
